# Association of biological age acceleration with cardiovascular disease and premature mortality: a population-based prospective cohort study

**DOI:** 10.3389/fpubh.2026.1816971

**Published:** 2026-05-19

**Authors:** Yingying Yang, Shaohua Yin, Dan Li, Huiqin Wan, Jia He, Lei Yuan, Wei Wang

**Affiliations:** 1 Clinical Research Unit, Shanghai First Maternity and Infant Hospital, Shanghai Key Laboratory of Maternal Fetal Medicine, Shanghai Institute of Maternal-Fetal Medicine and Gynecologic Oncology, School of Medicine, Tongji University, Shanghai, China; 2 Department of Medical Engineering, Peking University Third Hospital, Beijing, China; 3 Department of Cardiology and Institute of Vascular Medicine, Beijing Key Laboratory of Cardiovascular Receptors Research, Peking University Third Hospital, Beijing, China; 4 State Key Laboratory of Vascular Homeostasis and Remodeling, NHC Key Laboratory of Cardiovascular Molecular Biology and Regulatory Peptides, Peking University, Beijing, China; 5 School of Medicine, Tongji University, Shanghai, China; 6 Department of Military Health Statistics, Navy Medical University, Shanghai, China; 7 Department of Health Management, Naval Medical University, Shanghai, China; 8 Department of Gynecology, Shanghai First Maternity and Infant Hospital, Shanghai Key Laboratory of Maternal Fetal Medicine, Shanghai Institute of Maternal-Fetal Medicine and Gynecologic Oncology, School of Medicine, Tongji University, Shanghai, China

**Keywords:** biological, r disease, cohort study, premature agecardiovascula mortality, sex

## Abstract

**Introduction:**

Accelerated biological ageing is associated with age-related diseases, but sex differences in its association with cardiovascular disease (CVD) and premature mortality remain largely unknown. We aimed to assess the associations between biological age (BA) acceleration and CVD, premature mortality, and examine potential sex differences.

**Methods:**

This population-based prospective cohort study included participants aged 39 to 71 years from UK Biobank study, recruited between 2006 and 2010, and followed up until Dec 20, 2022. BA, derived from clinical biomarkers, was calculated using the Klemera-Doubal method (KDM-BA) and PhenoAge algorithms. BA acceleration was defined as the residual from regressing BA based on chronological age. Incident CVD and premature mortality (defined as death before age 70) were identified using ICD-9 and ICD-10 codes. Multivariable-adjusted Cox proportional hazards models were used to estimate the hazard ratios (HRs) and 95% confidence intervals (CIs) across BA acceleration quartiles.

**Results:**

Among 122,133 participants who were free of CVD at baseline (mean [SD] age, 56.0 [8.1] years; 65,442 [53.6%] women), 24,281 incident CVD cases and 3,614 premature deaths were reported. Restricted cubic splines showed progressively increasing risks of incident CVD and premature mortality associated with higher BA acceleration. Compared with the lowest quartile of KDM-BA acceleration, the largest adjusted HRs for incident CVD and premature mortality were 1.32 (95% CI 1.27–1.37) and 1.10 (95% CI 1.05–1.21) for quartile 4, respectively. For PhenoAge acceleration, the corresponding HRs were 1.23 (95% CI 1.19–1.28) and 1.21 (95% CI 1.10–1.33), respectively. These associations were more pronounced among male participants (*P*-interaction<0.05).

**Discussion:**

In this cohort study, higher BA acceleration was associated with increased risks of incident CVD and premature mortality, with more pronounced association observed in males. These findings suggest the need to exploring BA acceleration as a modifiable risk factor to optimize risk assessment, and to implement sex-specific strategies to improve health outcome.

## Introduction

Premature mortality is substantially declining in many countries, yet it remains a significant public health challenge (1, 2). Globally, 17 million people died from noncommunicable diseases (NCDs) before age 70, and cardiovascular disease (CVD), cancers, chronic respiratory diseases and diabetes account for over 80% of all premature NCD deaths (3). CVD is a predominant contributor to premature mortality, with the estimated global age-standardized premature mortality rate from total CVD of 96.04 per 100,000 people (4). Although more than 80% of these cases occur in low- and middle-income countries (5, 6), recent figures indicate nearly 174,594 deaths annually among approximately 6.4 million CVD people in the UK, including 48,662 under the age of 75 (7).

Increasing evidence suggests that genetic predispositions, environmental factors, and lifestyle behaviors are associated with CVD and CVD-related mortality (8–11). However, traditional risk factors may not fully reflect the complexity of CVD development. Biological age (BA), measured through clinical biomarkers, can be compared with chronological age to determine BA acceleration, indicating when an individual is biologically older (12). To comprehensively assess BA, we employed two validated measures: the Klemera-Doubal Method (KDM-BA) and PhenoAge. KDM-BA primarily reflects physiological aging and multisystem decline, whereas PhenoAge is more strongly associated with mortality risk. Using both measures allows for a complementary assessment of aging processes relevant to CVD and premature mortality. BA acceleration may reflect the cumulative effects of genetic predisposition, environmental exposures, and lifestyle behaviors, which is associated with age-related health outcomes, particularly CVD and mortality (13, 14). A recent multicenter longitudinal study of 913 participants, with 30 years of follow-up, found that BA acceleration was associated with 31% higher risks of CVD (15). A retrospective cohort study involving 557,940 Koreans aged 20–93 years showed that a 1-year increase in BA acceleration was associated with a 17.3% increased risk of mortality for men and a 13% increased risk for women (16).

Most of the existing studies have small sample sizes, lack evidence for sex-specific studies (17), and the extent to which BA acceleration is associated with premature mortality remains unclear. We focused on CVD as the primary outcome given its leading contributor to mortality among major NCDs globally, while premature mortality was included as a complementary endpoint capturing overall disease burden. This study aimed to investigate the association of BA acceleration with CVD and premature mortality, and to assess potential sex-specific difference, thereby addressing aging-related health patterns relevant to public health and preventive strategies.

## Materials and methods

### Study design and participants

The study protocol and baseline characteristics have been reported elsewhere (18). Briefly, the UK Biobank (UKB) cohort is a large, population-based prospective cohort study with over 500,000 participants aged 39–71 years recruited at 22 centers across England, Scotland, and Wales in the UK between 2006 and 2010. At baseline, participants completed a series of baseline assessments to provide detailed information about demographic characteristics, lifestyle behaviors, and health outcomes. Physical measurements, including blood pressure, and biological samples were collected.

### Assessment of BA and BA acceleration

BA was calculated using two validated algorithms—Klemera-Doubal method BA (KDM-BA) and PhenoAge—based on a series of clinical biomarkers. Both algorithms have been validated in multi-ethnic cohorts from China and UK for predicting disease, disability, and mortality (19, 20). The biomarkers used in computing two BAs, and their associations with cardiovascular disease (CVD) and premature mortality were reported (Supplementary Table 1). KDM-BA was derived from forced expiratory volume in one second (FEV1), systolic blood pressure, and seven blood chemistry parameters. PhenoAge was calculated from nine blood chemistry parameters, four of which overlap with those used in KDM-BA. BA was calculated using the R package ‘BioAge’ (https://github.com/dayoonkwon/BioAge). Individual differences in biological aging were assessed regressing BA on chronological age at biomarker measurement, and residuals were calculated. These residuals, referred to as BA acceleration, were considered as the measure of biological aging. To facilitate comparison between the two biological aging measures, we standardized the BA acceleration to have a mean of 0 and a standard deviation (SD) of 1 for continuous analysis, and categorized it into quartiles for dose–response analysis.

### Covariates assessment

Baseline sociodemographic variables, including age, sex, ethnicity, and education, were collected. The Townsend deprivation index, a composite measure of socioeconomic status, was calculated from participants’ postal codes and categorized into quintiles from 1 (least deprived) to 5 (most deprived). Lifestyle factors were assessed using validated questionnaires and included smoking status, alcohol consumption, physical activity, television viewing/sedentary time, sleep duration, and diet quality. Smoking status was classified as current (at risk) or past/never (not at risk). Alcohol consumption was classified as at risk for daily/almost daily consumption, and not at risk for ≤4 times/week. Physical activity was assessed using the IPAQ short form; <150 min/week moderate or <75 min/week vigorous activity was considered at risk. Television viewing/sedentary time was assessed by daily television viewing, with ≥4 h/day at risk. Sleep duration <7 or >9 h/day was classified as at risk, and 7–9 h/day as not at risk. Dietary quality was assessed through a validated questionnaire, and included fruit and vegetable intake (<400 g/day), oily fish (<1 portion/week), red meat (>3 portions/week), and processed meat (>1 portion/week); each scored as at risk if outside the recommended range. To assess overall lifestyle, each lifestyle factor was scored as either 0 (not at risk) or 1 (at risk), resulting in an unweighted lifestyle score ranging from 0 to 9, with higher scores indicating less healthy lifestyle. Participants were then classified into three groups based on their lifestyle score: most healthy (scores of 0–2), moderately healthy (scores of 3–5), and least healthy (scores of 6–9) (21). Body mass index (BMI) was calculated from measured height and weight, and participants were categorized as underweight, normal weight, overweight, or obese according to World Health Organization criteria. The Charlson Comorbidity Index (CCI), which assesses comorbidities using International Classification of Diseases, Tenth Revision (ICD-10) diagnosis codes, was calculated and categorized as 0 (no comorbidity), 1 (one comorbidity), and ≥2 (two or more comorbidities).

### Outcome ascertainment

Outcomes included baseline cardiovascular disease (CVD), incident CVD during follow-up, and premature mortality. Baseline CVD was defined using self-reports diagnoses of angina, stroke, hypertension, and heart attack based on prior study (22). Incident CVD included coronary heart disease, stroke, definite angina, heart failure, peripheral artery disease, coronary artery bypass graft, identified using hospital records using ICD-9 and ICD-10 codes (Supplementary Table 2). For example, coronary heart disease was identified using ICD-10 codes I20–I25 [except I24.1] and I46, and ICD-9 codes 36.0–36.2, 410, 411, 413, 414, 4,149, 4,275, and 4,100–4,109 (23). Incident CVD was additionally determined from cardiovascular mortality using ICD-10 codes I00-I99, obtained from national death registry data. All-cause and cause-specific mortality, classified by underlying cause of death, were recorded in national death registries. Causes of death were CVD (I00-I99), cancer (C00-D48), respiratory diseases (J00-J99), and other causes, including external causes such as accidents or injuries (24–26). Premature mortality was defined as death before age 70 (24, 27). Participants aged 70 or older at death, or those remaining event-free at the end of the study, were censored in the analysis.

### Ethics approval and consent to participate

This study was approved by the UK North West Multi-centre Research Ethics Committee (Ref 11/NW/0382 on June 14, 2011), and all participants provided written informed consent.

### Statistical analysis

Descriptive statistics were used to summarize the individual baseline characteristics. Categorical variables were presented as frequencies and percentages, and continuous variables as means with standard deviations (SDs). Incidence rates for CVD and premature mortality were calculated based on the number of participants with CVD or deaths, expressed per 100 individuals or per 1,000 person-years of follow-up, as appropriate.

**Figure 1:**
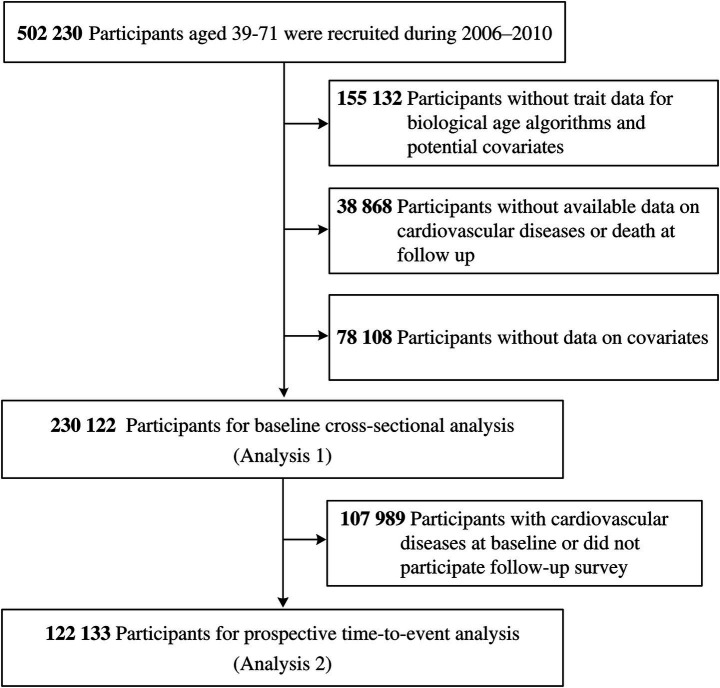
Flowchart for the selection of study participants.

Associations of BA acceleration with incident CVD and premature mortality were assessed using restricted cubic spline regression models, with knots at the 5th, 50th, and 95th percentiles. Nonlinearity was assessed by likelihood ratio tests comparing spline models with linear models. All models were adjusted for the covariates as in the primary analyses. Logistic regression was used to assess associations of BA acceleration with baseline CVD (Analysis 1), and Cox proportional hazards model were used for incident CVD and premature mortality (Analysis 2). Proportional hazards assumption was evaluated graphically using log cumulative hazard plot, with the log(time) plotted against the estimated log cumulative hazard for the exposure. A sequence of regression models was performed: (1) adjusted for age, sex, ethnic, education, Townsend deprivation index quintile, and BMI; (2) further adjusted for Charlson Comorbidity Index; and (3) additionally adjusted for lifestyle score. The effect estimates are expressed per standard deviation increase. Observations with missing values were excluded from this analysis (Figure 1).

Sex differences were assessed by including interaction terms between sex and BA acceleration in multivariable models for incident CVD and premature mortality.

Three sensitivity analyses were performed. First, participants in Analysis 2 with ≤2 years of follow-up were excluded to reduce reversal causation. Second, models were additionally adjusted for cancer diagnoses during follow-up to account for potential mediation. Third, missing covariate data were addressed using multiple imputation approach with five imputations.

All statistical analyses were done with SAS version 9.4 (SAS Institute Inc.) and Stata version 12. A two-sided *p*-value of less than 0.05 was considered statistically significant.

### Results

**Figure 2:**
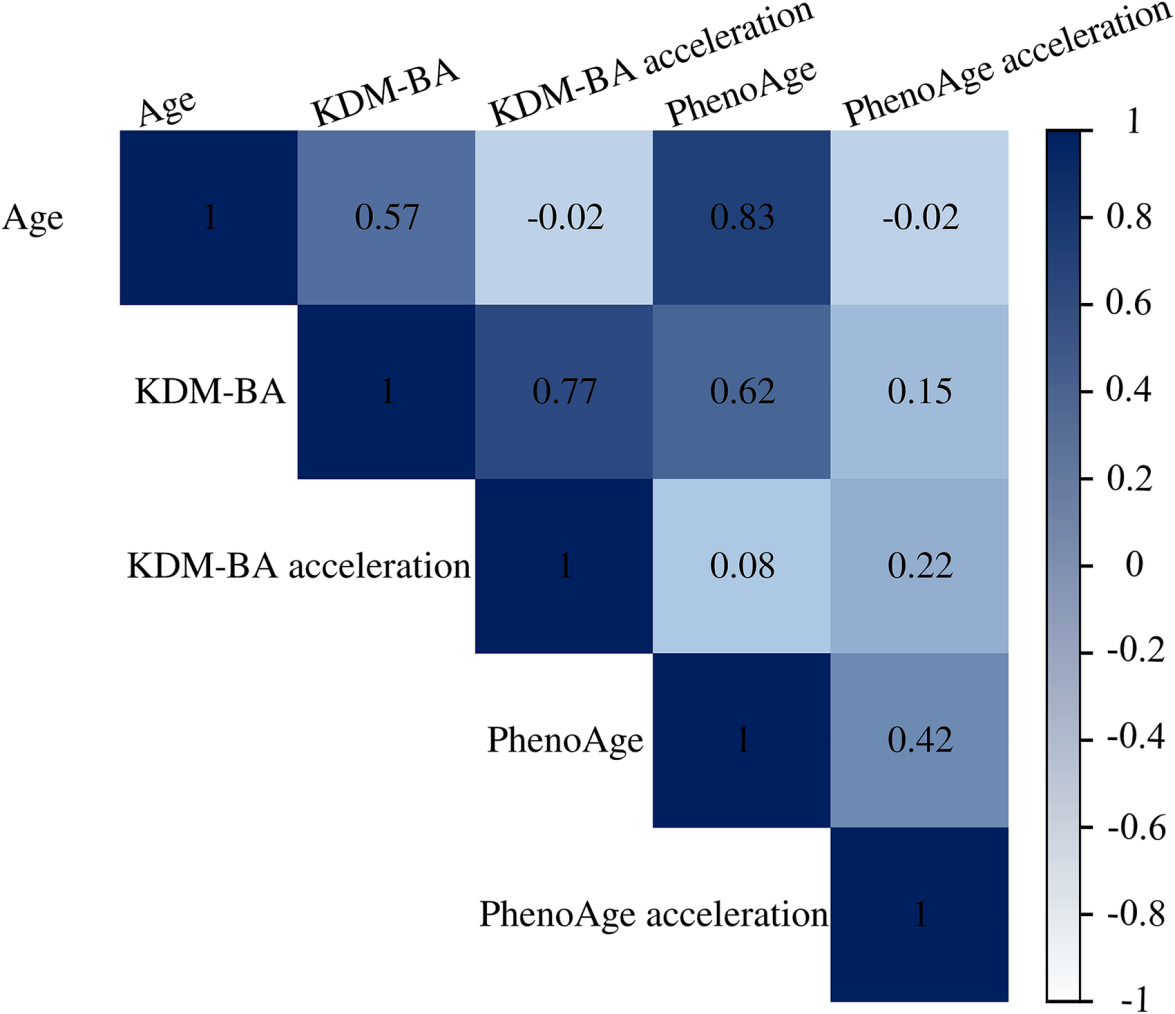
Correlation matrix of chronological age, biological age, and biological age acceleration (Pearson correlation).

A total of 230,122 participants with available data on estimating BA and completed health-related surveys were included in the analysis. To minimize reverse causation, 122,133 participants who were free of baseline CVD and with follow-up data were further analyzed (Figure 1). Baseline characteristics of participants included in the two analyses are shown in Table 1. Mean (SD) age and BMI were 56.0 (8.1) years and 27.0 (4.6) kg/m^2^, respectively. Almost all participants were White, 46.2% were male, 12–13% had college or university education, baseline diabetes prevalence ranged from 2.2 to 8.4%, and baseline cancer prevalence was 17.5–18.1%. Baseline CVD was present in 28.6%. Among participants free of baseline CVD, incidence rates were 23.0 and 2.0 per 1,000 person-years for CVD and premature mortality, respectively, over a median of follow-up of 9 years. Baseline BA was positively correlated with chronological age, and BA acceleration showed a moderate correlation (Pearson *r* = 0.2) (Figure 2).

**Table 1:** Characteristics of study participants^a^.

Characteristic	Analysis 1 (*N* = 230,122)	Analysis 2 (*N* = 122,133)
Gender		
Female	120,542 (52.4)	65,442 (53.6)
Male	109,580 (47.6)	56,691 (46.4)
Age group		
≤54	89,834 (39.0)	52,117 (42.7)
55–59	41,513 (18.0)	22,390 (18.3)
60–64	56,164 (24.4)	28,307 (23.2)
≥65	42,611 (18.5)	19,319 (15.8)
Ethnicity		
White	221,039 (96.1)	117,611 (96.3)
Non-white	9,083 (3.9)	4,522 (3.7)
Education		
College or University degree	28,351 (12.3)	15,777 (12.9)
Others	201,771 (87.7)	106,356 (87.1)
Townsend deprivation index quintile		
1 (least deprived)	46,016 (20.0)	25,219 (20.6)
2	45,864 (19.9)	24,879 (20.4)
3	46,140 (20.1)	24,625 (20.2)
4	46,016 (20.0)	24,134 (19.8)
5 (most deprived)	46,086 (20.0)	23,276 (19.1)
Smoking status		
Never	125,278 (54.4)	68,051 (55.7)
Previous	82,202 (35.7)	41,273 (33.8)
Current	22,642 (9.8)	12,809 (10.5)
Alcohol drinking		
Never	214,514 (93.2)	114,552 (93.8)
Previous	8,186 (3.6)	4,104 (3.4)
Current	7,422 (3.2)	3,477 (2.8)
Body mass index at recruitment (kg/m^2^)		
<18.5	1,066 (0.5)	643 (0.5)
18.5–24.9	76,113 (33.1)	46,192 (37.8)
25–29.9	99,768 (43.4)	53,125 (43.5)
≥30	53,175 (23.1)	22,173 (18.2)
Cardiovascular disease		
No	164,223 (71.4)	122,133 (100)
Yes	65,899 (28.6)	0
Lifestyle score		
Most healthy (0–2)	137,104 (59.6)	74,085 (60.7)
Moderately healthy (3–5)	88,011 (38.2)	45,516 (37.3)
Least healthy (6–9)	5,007 (2.2)	2,532 (2.1)
Charlson comorbidity index		
0	131,043 (56.9)	71,772 (58.8)
1	64,292 (27.9)	34,182 (28.0)
≥2	34,787 (15.1)	16,179 (13.2)
Diabetes		
No	210,713 (91.6)	119,449 (97.8)
Yes	19,409 (8.4)	2,684 (2.2)
Hypertension		
No	174,458 (75.8)	122,133 (100)
Yes	55,664 (24.2)	0
Cancer		
No	189,908 (82.5)	100,011 (81.9)
Yes	40,214 (17.5)	22,122 (18.1)
Biological ages		
KDM-BA	52.9 (12.33)	51.24 (12.03)
KDM-BA acceleration	−0.05 (1.00)	−0.15 (0.97)
PhenoAge	50.11 (9.2)	49.15 (8.95)
PhenoAge acceleration	−0.01 (1.00)	−0.07 (0.96)
Components of biological age		
Forced expiratory volume in 1 s (L)^*^	2.77 (0.77)	2.82 (0.77)
Systolic blood pressure (mm Hg)^*^	137.27 (18.27)	134.57 (17.36)
Total Cholesterol (mg/dL)^*^	220.23 (43.52)	225.76 (41.49)
Glycated hemoglobin (%)^*^	5.42 (0.54)	5.36 (0.46)
Blood urea nitrogen (mg/dL)^*^	15.11 (3.64)	14.87 (3.43)
Lymphocyte (%)^#^	28.95 (7.26)	29.18 (7.2)
Mean cell volume (fL)^#^	82.84 (5.2)	82.95 (5.18)
Serum glucose (mg/dL)^#^	91.42 (18.73)	89.8 (16.06)
Red cell distribution width (%)^#^	13.45 (0.88)	13.44 (0.88)
White blood cell count (1,000 cells/uL)^#^	6.81 (1.72)	6.7 (1.68)
Albumin (g/dL)^*#^	4.53 (0.26)	4.52 (0.25)
Creatinine (mg/dL)^*#^	0.82 (0.17)	0.81 (0.16)
C-reactive protein (mg/dL)^*#^	0.24 (0.36)	0.22 (0.34)
Alkaline phosphatase (U/L)^*#^	82.13 (22.56)	81.16 (22.17)

^a^Included overall 230,122 participants for Analysis 1, the subgroup of 122,133 participants who were free of cardiovascular disease at baseline and completed follow-up survey data for Analysis 2; Mean values (standard deviation) for continuous variables and *n* (%) for categorical variables; percentages may not sum to 100 due to rounding.

*Used to calculate biological age by using the Klemera-Doubal method.

^#^Used to calculate PhenoAge algorithm.

Higher KDM-BA acceleration and PhenoAge acceleration were associated with increased odds of baseline CVD (Table 2). Adjustment for Charlson Comorbidity Index did not substantially change these associations, and further adjustment for lifestyle score attenuated them but remaining statistically significant. Similar monotonic increase were observed when BA acceleration was classified into quartiles. In the fully adjusted model, for example, the ORs for baseline CVD according to the quartiles of KDM-BA acceleration were 1.27 (95% confidence interval [CI] 1.23–1.31) for Q2, 1.56 (95% CI 1.51–1.61) for Q3, and 2.11 (95% CI 2.05–2.17) for Q4, compared to Q1.

**Table 2:** Associations of the biological age (KDM-BA and PhenoAge) accelerations with the baseline CVD^a^.

BA		aOR (95% CI)		
	Event/N	Model 1^a^	Model 2^b^	Model 3^c^
KDM-BA acceleration (Continuous)	65,899/230122	**1.39 (1.38–1.41)**	**1.35 (1.34–1.36)**	**1.35 (1.33–1.36)**
KDM-BA acceleration (Quartiles)				
Q1	11,854/57292	Ref	Ref	Ref
Q2	14,301/58167	**1.30 (1.26–1.33)**	**1.27 (1.23–1.31)**	**1.27 (1.23–1.31)**
Q3	17,098/57076	**1.62 (1.57–1.67)**	**1.56 (1.52–1.61)**	**1.56 (1.51–1.61)**
Q4	22,646/57587	**2.28 (2.22–2.35)**	**2.12 (2.06–2.19)**	**2.11 (2.05–2.17)**
PhenoAge acceleration (Continuous)	65,899/230122	**1.16 (1.14–1.17)**	**1.11 (1.10–1.12)**	**1.10 (1.09–1.11)**
PhenoAge acceleration (Quartiles)				
Q1	13,485/57946	Ref	Ref	Ref
Q2	14,839/56677	**1.03 (1.00–1.06)**	**1.02 (0.99–1.05)**	**1.01 (0.99–1.04)**
Q3	16,780/58153	**1.08 (1.05–1.11)**	**1.04 (1.01–1.07)**	**1.04 (1.01–1.07)**
Q4	20,795/57346	**1.36 (1.32–1.40)**	**1.23 (1.20–1.27)**	**1.22 (1.18–1.25)**

KDM-BA, Klemera-Doubal Method biological age; aOR, adjusted odds ratio; 95% CI, 95% confidence interval; CVD, cardiovascular disease; Q, quartile. KDM-BA and PhenoAge acceleration were standardized (mean = 0, SD = 1) and odds ratios for continuous analyses represent the effect per 1-standard deviation increase in biological age acceleration.

^a^Model 1 was adjusted for age, sex, ethnic, education, Townsend deprivation index quintile, and body mass index.

^b^Model 2 was adjusted for Model 1 covariates and Charlson Comorbidity Index.

^c^Model 3 was adjusted for Model 2 covariates lifestyle score. Two-sided statistical tests were conducted and bolded values are statistically significant (<0.05).

**Figure 3:**
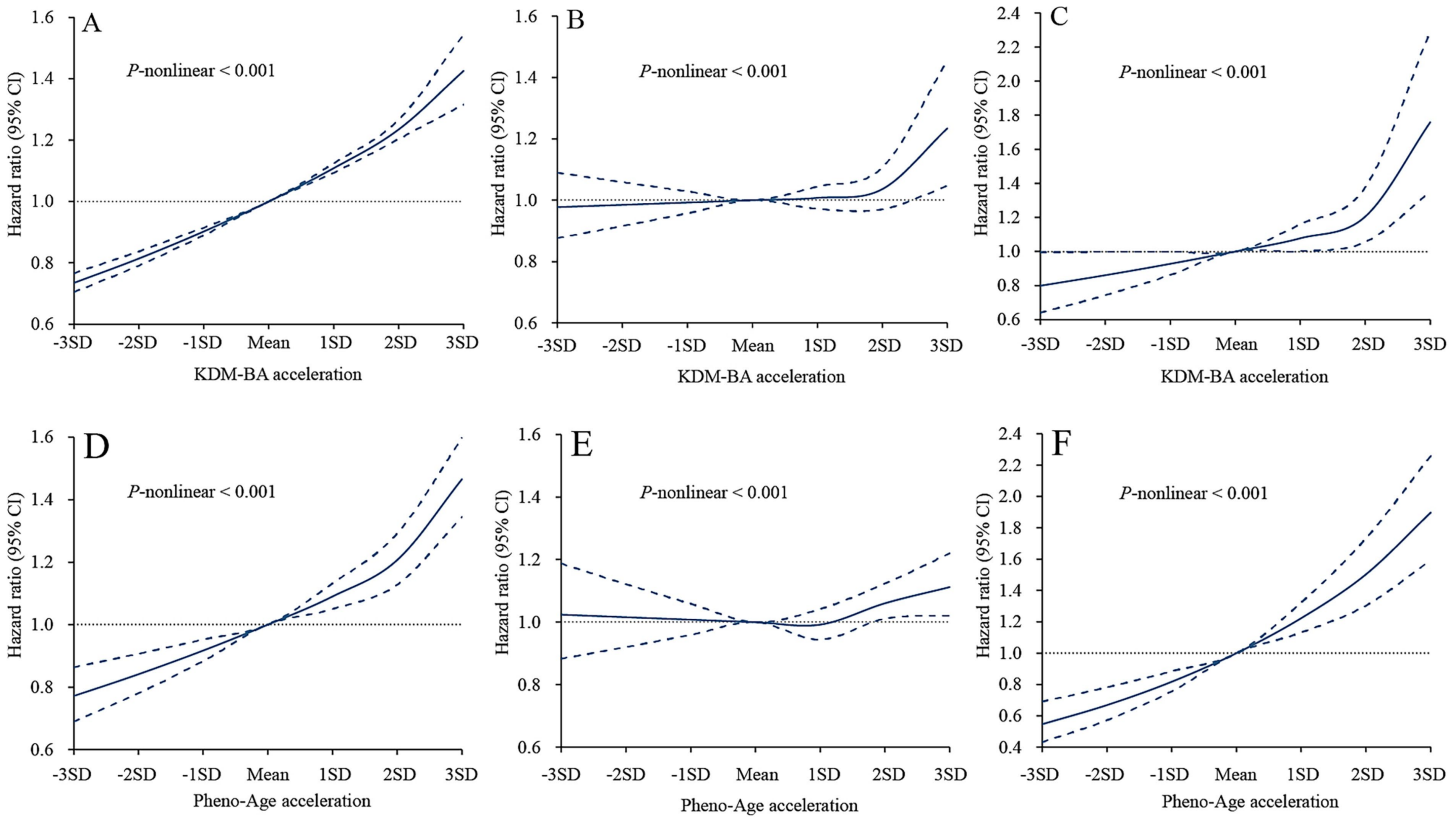
Relationships of KDM-BA acceleration and PhenoAge acceleration with incident CVD, all-cause premature mortality, and premature mortality from CVD. **(A)** KDMBA acceleration with incident CVD, **(B)** KDMBA acceleration with all-cause premature mortality, **(C)** KDMBA acceleration with premature death from CVD, and **(D)** PhenoAge acceleration with incident, **(E)** PhenoAge acceleration with all-cause premature mortality, **(F)** PhenoAge acceleration with premature death from CVD. Solid line: Point estimation; Dash line: Confidence limits. Restricted cubic spline regression model adjusted for age, sex, ethnicity, education, Townsend deprivation index quintile, BMI, Charlson Comorbidity Index, and lifestyle score.

**Figure 4:**
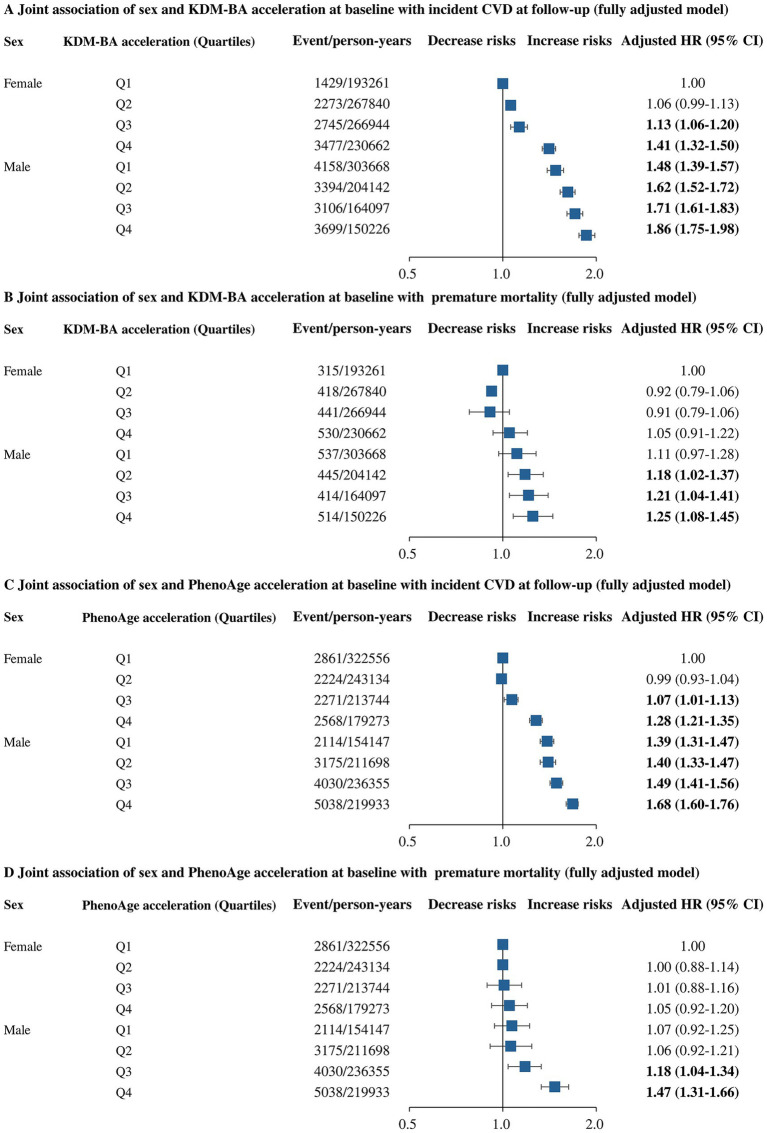
Association of KDM-BA acceleration, PhenoAge acceleration, and sex with incident CVD, all-cause premature mortality. **(A)** KDMBA acceleration, sex with incident CVD, **(B)** KDMBA acceleration, sex with all-cause premature mortality, **(C)** PhenoAge acceleration, sex with incident CVD, and **(D)** PhenoAge acceleration, sex with all-cause premature mortality. Solid line: Point estimation; Dash line: Confidence limits. Restricted cubic spline regression model adjusted for age, sex, ethnicity, education, Townsend deprivation index quintile, BMI, Charlson Comorbidity Index, and lifestyle score.

Dose–response analysis showed different patterns of relationships of BA acceleration with incident CVD and premature mortality, with all *P* for overall association or nonlinearity <0.001 (Figure 3). The risks of incident CVD and premature mortality from CVD increased monotonically with higher BA acceleration. For all-cause premature mortality, risks initially plateaued up to 1SD of BA acceleration, and then accelerated. After adjustment for age, sex, ethnicity, education, Townsend deprivation index quintile, BMI, and Charlson Comorbidity Index, KDM-BA acceleration remained associated with increased risks of incident CVD and all-cause premature mortality (Supplementary Table 3). Further adjustment for lifestyle score attenuated these associations. For incident CVD, the HRs across quartiles of KDM-BA acceleration were 1.08 (95% CI 1.04–1.12) for Q2, 1.14 (95% CI 1.10–1.18) for Q3, and 1.32 (95% CI 1.27–1.37) for Q4, compared to Q1 (Table 3); for all-cause premature mortality, the HRs were 1.00 (95% CI 0.91–1.10), 1.01 (95% CI 0.92–1.11), 1.10 (95% CI 1.05–1.21), respectively. Similar results were observed for the PhenoAge acceleration, with slightly higher HRs for incident CVD and somewhat lower HRs for all-cause premature mortality (Table 3). To examine the joint effect of BA acceleration quartile and sex on incident CVD and premature mortality, participants were categorized into eight groups according to BA acceleration quartile and sex (Figure 4). Compared with females in Q1 of KDM-BA acceleration, males in Q4 had the highest risks of incident CVD (HR 1.86, 95% CI 1.75–1.98) and premature mortality (HR 1.25, 95% CI 1.08–1.45). Similarly, males in Q4 of PhenoAge acceleration had the highest risks of incident CVD (HR 1.68, 95% CI 1.60–1.76) and premature mortality (HR 1.47, 95% CI 1.31–1.66) relative to females in Q1. Within each sex category, higher BA acceleration quartiles were associated with higher risk of both outcomes (Figure 4). In cause-specific premature mortality analyses, higher BA acceleration was not associated with premature death from cancer, but was associated with higher risk of premature death from CVD, respiratory diseases and of all other causes (Supplementary Table 4, Supplementary Figure 1). Significant interactions between BA acceleration and male sex were observed for incident CVD and premature mortality (*P* for interaction<0.001), except for KDM-BA acceleration and sex on premature mortality (*P* for interaction = 0.596) (Supplementary Table 5).

**Table 3:** Associations of biological age (KDM-BA and PhenoAge) with incident CVD and premature mortality at follow-up (fully adjusted model)^a^.

BA	Incident CVD		Premature mortality	
	Event/person-years	aHR (95% CI)	Event/person-years	aHR (95% CI)
KDM-BA acceleration (continuous)	24,281/1057040	**1.11 (1.10–1.13)**	3614/1780840	**1.02 (1.01–1.06)**
KDM-BA acceleration (quartiles)				
Q1	5587/294957	Ref	852/496929	Ref
Q2	5667/279005	**1.08 (1.04–1.12)**	863/471982	1.00 (0.91–1.10)
Q3	5851/255330	**1.14 (1.10–1.18)**	855/431041	1.01 (0.92–1.11)
Q4	7176/227748	**1.32 (1.27–1.37)**	1044/380888	**1.10 (1.05–1.21)**
PhenoAge acceleration (Continuous)	24,281/1057040	**1.11 (1.09–1.12)**	3614/1780840	**1.13 (1.10–1.17)**
PhenoAge acceleration (quartiles)				
Q1	4975/281933	Ref	746/476703	Ref
Q2	5399/270618	1.00 (0.96–1.04)	757/454832	0.98 (0.88–1.08)
Q3	6301/266967	**1.07 (1.03–1.11)**	894/450099	1.04 (0.94–1.15)
Q4	7606/237522	**1.23 (1.19–1.28)**	1217/399206	**1.21 (1.10–1.33)**

KDM-BA, Klemera-Doubal Method biological age; aHR, adjusted hazard ratio; 95% CI, 95% confidence interval; CVD, cardiovascular disease; Q, quartile.

^a^Model was adjusted for age, sex, ethnic, education, Townsend deprivation index quintile, body mass index, Charlson Comorbidity Index, lifestyle score. Two-sided statistical tests were conducted and bolded values are statistically significant (<0.05). KDM-BA and PhenoAge acceleration were standardized (mean = 0, SD = 1) and hazard ratios for continuous analyses represent the effect per 1-standard deviation increase in biological age acceleration.

Several sensitivity analyses were conducted. First, restricting the sample to participants with >2 years of follow-up, higher BA acceleration quartiles remained associated with increased risks of incident CVD and premature mortality (Supplementary Table 6). Second, to account for mediation by cancer incidence, additional adjustment for cancer during follow-up was conducted, which did not materially change the associations (Supplementary Table 7). Third, to address potential biases from missing data, multiple imputation by chained equations was used, yielding results consistent with the complete-case analysis (Supplementary Table 8).

## Discussion

In this large, population-based cohort study, we assessed the associations of BA acceleration, measured by KDM-BA and PhenoAge, with CVD and premature mortality. Our study showed that higher BA acceleration, whether analyzed as a continuous or categorical variable, was associated with higher odds of baseline CVD and increased hazard of incident CVD and premature mortality. Furthermore, the associations appeared stronger in males than in females, suggesting potential sex-specific differences.

A recent study from the UKB cohort (28) reported that KDM-BA and PhenoAge accelerations were associated with higher risk for cardiometabolic multimorbidity and long-term mortality. Another study of Chinese adult cohort, based on the China Kadoorie Biobank (29), indicated that KDM-BA was associated with the long-term mortality risk, particularly among participants at different stages of the CVD. Moreover, a national Mendelian randomisation study using data from the National Health and Nutrition Examination Survey (30), showed that PhenoAge was associated with several cardiovascular outcomes, including atrial fibrillation, myocardial infarction, coronary heart disease, ischemic heart disease, and small vessel stroke. A retrospective cohort study of 557,940 Koreans examined the sex differences in the effects of BA acceleration on mortality (16), showing that a 1-year increase in BA corresponded to 17.3% higher risk in men and 13.0% higher risk in women. Although these findings are consistent with our results, several studies reported inconsistent relationships of BA and CVD and mortality (15, 31). A multicenter longitudinal study from the Coronary Artery Risk Development in Young Adults cohort, following 913 Black and White participants for 20 years, found that higher KDM-BA was associated with significantly higher risk of incident CVD, while higher PhenoAge acceleration was associated with lower risk of incident CVD (15). Another longitudinal study using data from the SardiNIA and InCHIANTI cohorts (31), showed that the KDM biological aging was not significantly associated with mortality. Further research is needed to investigate the potential influences of population characteristics, study designs, and methodological differences on these associations.

Several potential mechanisms may underlie the observed associations between BA acceleration and health outcomes, including physiological dysregulation and genetic predisposition (8). First, BA acceleration likely reflects cumulative physiological dysregulation, including chronic activation of inflammatory pathways and oxidative stress, both implicated in atherosclerosis and CVD development (32). Although these processes were not directly measured, prior evidence suggests that circulating inflammatory biomarkers included in BA algorithms, such as C-reactive protein, may partially reflect systemic inflammatory burden related to aging-related disease risk (33, 34). Second, although epigenetic regulation and telomere attrition have been implicated in biological aging and age-related functional decline (35, 36), their relevance to BA acceleration in the present study requires further validation. These factors may act synergistically to increase cardiovascular risk through long-term exposure to adverse metabolic states. The stronger association observed in men may reflect several mechanisms (37). First, men are more prone to X-linked recessive conditions and present age-associated loss of the Y chromosome in blood cells, linking to genomic instability and increasing disease susceptibility (38). Second, hormones such as estrogen, predominantly in women, provided protective cardiovascular effects through anti-inflammatory properties and beneficial lipid metabolism, whereas lower estrogen levels in men may be associated with increased susceptibility to the cardiovascular effects of BA acceleration (39). Additionally, men are more prone to immunosenescence and inflammaging, processes associated with age-related diseases including CVD, diabetes, and neurodegeneration (40).

Strengths of this study included a large sample size, comprehensive clinical biomarkers data, and long-term follow-up of all participants. In addition, using two validated BA algorithms (KDM-BA and PhenoAge) allows a comprehensive analysis of CVD and premature mortality related to biological aging. This study has limitations. First, these associations might not indicate causal relationship owing to observational study design. However, in participants free of CVD at baseline, higher BA was associated with incident CVD and premature mortality during follow-up, which lasted over 2 years, based on prospective analysis. Second, BA calculated from a single baseline measurement of clinical biomarkers did not account for temporal changes, potentially underestimating associations and limiting causal inferences. Third, the study population was a volunteer cohort, predominantly of White ethnicity, who were likely healthier and wealthier, potentially limiting generalizability. Fourth, although adjustment for multiple confounders, residual confounding from unmeasured variables may remain. Fifth, the large cohort and reliance on diagnostic coding may introduce unavoidable errors and omissions, resulting in underreporting and nondifferential misclassification that could bias the associations toward the null. Sixth, self-reported baseline CVD and registry-based incident CVD may introduce differential misclassification, and including hypertension at baseline but not as an incident CVD may cause some asymmetry in disease definitions.

## Conclusion

In this large, population-based cohort, higher BA acceleration was associated with higher risks of CVD and premature mortality, with more pronounced association observed in men. These findings suggest that BA may serve as a potential risk marker for improved risk stratification, and highlight the importance of considering sex-specific differences in future preventive strategies.

## Data Availability

The data analyzed in this study is subject to the following licenses/restrictions: Access to data from UK Biobank (https://www.ukbiobank.ac.uk/) is available upon application. Requests to access these datasets should be directed to 2405132@tongji.edu.cn.
