# Supplementary material for: Association of biological age acceleration with cardiovascular disease and premature mortality: a population-based prospective cohort study
Source: Front Public Health. 2026 May 19;14:1816971. doi: 10.3389/fpubh.2026.1816971 (PMC13226196; doi:10.3389/fpubh.2026.1816971)
Supplement: Supplementary file 1 [file Data_Sheet_1.DOCX]

**Association of biological age acceleration with cardiovascular disease and premature mortality: A population-based prospective cohort study**

**Table S1 Association between biomarkers and biomarkers used in calculating biological age and incident CVD and premature mortality**

|  | Baseline CVD | Incident CVD | Premature mortality |
| --- | --- | --- | --- |
| **Biomarkers** | OR (95% CI) | HR (95% CI) | HR (95% CI) |
| Forced expiratory volume in one second (L) | 0.75 (0.74-0.76) | 0.86 (0.85-0.88) | 0.98 (0.94-1.02) |
| Systolic blood pressure (mm Hg) | 1.04 (1.04-1.04) | 1.01 (1.01-1.02) | 1.00 (1.00-1.00) |
| Total Cholesterol (mg/dL) | 0.99 (0.99-0.99) | 1.00 (1.00-1.00) | 1.00 (1.00-1.00) |
| Glycated hemoglobin (%) | 2.23 (2.19-2.27) | 1.80 (1.78-1.82) | 1.15 (1.08-1.22) |
| Blood urea nitrogen (mg/dL) | 1.08 (1.08-1.09) | 1.03 (1.03-1.04) | 0.96 (0.96-0.97) |
| Lymphocyte (%) | 0.98 (0.98-0.98) | 0.99 (0.99-0.99) | 0.98 (0.98-0.99) |
| Mean cell volume (fL) | 0.99 (0.99-0.99) | 1.00 (1.00-1.00) | 1.03 (1.02-1.04) |
| Serum glucose (mg/dL) | 1.02 (1.02-1.02) | 1.01 (1.01-1.01) | 1.00 (1.00-1.00) |
| Red cell distribution width (%) | 1.08 (1.07-1.09) | 1.11 (1.10-1.12) | 1.18 (1.14-1.21) |
| White blood cell count (1000 cells/uL) | 1.16 (1.16-1.17) | 1.11 (1.10-1.11) | 1.12 (1.10-1.14) |
| Albumin (g/dL) | 1.05 (1.02-1.09) | 0.68 (0.65-0.72) | 0.78 (0.69-0.89) |
| Creatinine (mg/dL) | 4.85 (4.59-5.12) | 2.50 (2.32-2.70) | 0.98 (0.79-1.21) |
| C-reactive protein (mg/dL) | 1.61 (1.57-1.64) | 1.41 (1.37-1.45) | 1.50 (1.41-1.60) |
| Alkaline phosphatase (U/L) | 1.01 (1.01-1.01) | 1.01 (1.01-1.01) | 1.01 (1.01-1.01) |

Abbreviations: CVD, cardiovascular disease; OR, odds ratio; HR, hazard ratio; 95% CI, 95% confidence interval.

**Tble S2** **Definitions of cardiovascular outcomes**

|  | Definitions from ICD-10 | Definitions from ICD-9 |
| --- | --- | --- |
| Cardiovascular Mortality | I00-I99 |  |
| Coronary Heart Disease | I20, I21, I22, I23, I24 (except I24.1), I25, I46 | 36.0, 36.1, 36.2, 410, 411, 413, 414, 4149, 4275, 4100, 4101, 4102, 4103, 4104, 4105, 4106, 4107, 4108, 4109 |
| Stroke | I60, I62, I61, I63 (except I63.6), I64, I65, I66, I67, I68, I69, G45 | 430, 431, 432, 433, 434, 435, 436, 437, 438 |
| Definite angina | I20 | 410 |
| Heart Failure | I11.0, I13.0, I13.2, I50, I51, I52 | 402, 428, 4021, 4029, 4040, 4040, 4041, 4049 |
| Peripheral artery disease | I70, I73, I74, E10, E11, E12, E13, E14.5 | 50.7, 440.2, 440.4, 443.8, 443.9, 444.2, 444.81 |
| Coronary artery bypass graft | I25 |  |

**Table S3** **Associations of biological age** **(KDM-BA and PhenoAge) with incident CVD and premature mortality at follow-up (models 1 & 2)**

|  | Incident CVD | | Premature mortality | |
| --- | --- | --- | --- | --- |
|  | Model 1^a^ | Model 2^b^ | Model 1^a^ | Model 2^b^ |
| KDM-BA acceleration (Continuous) | **1.22 (1.21-1.24)** | **1.12 (1.10-1.13)** | **1.22 (1.18-1.27)** | **1.04 (1.01-1.08)** |
| KDM-BA acceleration (Quartiles) |  |  |  |  |
| Q1 | Ref | Ref | Ref | Ref |
| Q2 | **1.15 (1.11-1.20)** | **1.08 (1.04-1.12)** | **1.12 (1.02-1.24)** | 1.02 (0.93-1.12) |
| Q3 | **1.28 (1.23-1.33)** | **1.14 (1.10-1.19)** | **1.24 (1.13-1.37)** | 1.04 (0.94-1.14) |
| Q4 | **1.65 (1.59-1.72)** | **1.33 (1.28-1.38)** | **1.68 (1.53-1.85)** | **1.15 (1.05-1.27)** |
| PhenoAge acceleration (Continuous) | **1.21 (1.20-1.23)** | **1.11 (1.10-1.12)** | **1.36 (1.32-1.41)** | **1.15 (1.11-1.18)** |
| PhenoAge acceleration (Quartiles) |  |  |  |  |
| Q1 | Ref | Ref | Ref | Ref |
| Q2 | **1.04 (1.00-1.08)** | 1.00 (0.96-1.04) | 1.03 (0.93-1.14) | 0.99 (0.89-1.09) |
| Q3 | **1.17 (1.13-1.22)** | **1.08 (1.04-1.12)** | **1.21 (1.09-1.33)** | 1.06 (0.96-1.17) |
| Q4 | **1.53 (1.48-1.59)** | **1.24 (1.20-1.29)** | **1.83 (1.67-2.01)** | **1.26 (1.15-1.38)** |

Abbreviations: KDM-BA, Klemera-Doubal Method biological age; aHR, adjusted hazard ratio; 95% CI, 95% confidence interval; CVD, cardiovascular disease; Q, quartile.

^a^Model 1 was adjusted for age, sex, ethnic, education, Townsend deprivation index quintile, and body mass index.

^b^Model 2 was adjusted for Model 1 covariates and Charlson Comorbidity Index. Two-sided statistical tests were conducted and bolded values are statistically significant (<0.05).

**Table S4** **Associations of biological age (KDM-BA and PhenoAge)** **acceleration with specific-cause premature mortality (fully adjusted model)^a^**

|  | Cardiovascular mortality | | Respiratory mortality | | Cancer-related mortality | | Other-cause mortality | |
| --- | --- | --- | --- | --- | --- | --- | --- | --- |
|  | Event/person-years | aHR (95% CI) | Event/person-years | aHR (95% CI) | Event/person-years | aHR (95% CI) | Event/person-years | aHR (95% CI) |
| KDM-BA acceleration (Continuous) | 860/1758634 | **1.12 (1.05-1.20)** | 321/1753597 | **1.28 (1.15-1.43)** | 2076/1767382 | 0.94 (0.90-1.01) | 357/1754140 | **1.11 (1.00-1.23)** |
| KDM-BA acceleration (Quartiles) |  |  |  |  |  |  |  |  |
| Q1 | 190/491670 | Ref | 64/490519 | Ref | 513/494023 | Ref | 85/490753 | Ref |
| Q2 | 186/466371 | 1.03 (0.84-1.26) | 66/465341 | 1.10 (0.78-1.56) | 534/469135 | 0.98 (0.87-1.11) | 77/465469 | 0.98 (0.72-1.34) |
| Q3 | 210/425862 | 1.19 (0.97-1.46) | 67/424473 | 1.18 (0.83-1.67) | 475/427676 | 0.87 (0.77-1.00) | 103/424871 | **1.44 (1.07-1.94)** |
| Q4 | 274/374731 | **1.33 (1.09-1.62)** | 124/373264 | **2.00 (1.46-2.75)** | 554/376548 | 0.91 (0.80-1.03) | 92/373047 | 1.33 (0.97-1.81) |
| PhenoAge acceleration (Continuous) | 860/1758634 | **1.25 (1.17-1.33)** | 321/1753597 | **1.40 (1.28-1.53)** | 2076/1767382 | 1.02 (0.98-1.06) | 357/1754140 | **1.35 (1.23-1.48)** |
| PhenoAge acceleration (Quartiles) |  |  |  |  |  |  |  |  |
| Q1 | 143/471682 | Ref | 44/470826 | Ref | 479/474343 | Ref | 80/471157 | Ref |
| Q2 | 166/450075 | 1.00 (0.80-1.26) | 56/449013 | **1.27 (0.85-1.89)** | 469/452298 | 0.98 (0.86-1.11) | 66/449198 | 0.81 (0.58-1.12) |
| Q3 | 221/444590 | 1.17 (0.94-1.44) | 81/443171 | **1.66 (1.14-2.40)** | 509/446662 | 0.96 (0.85-1.09) | 83/443272 | 0.96 (0.70-1.31) |
| Q4 | 330/392287 | **1.50 (1.22-1.84)** | 140/390587 | **2.42 (1.70-3.44)** | 619/394079 | 0.99 (0.87-1.12) | 128/390513 | **1.42 (1.06-1.91)** |

Abbreviations: KDM-BA, Klemera-Doubal Method biological age; aHR, adjusted hazard ratio; 95% CI, 95% confidence interval; Q, quartile.

^*^Model was adjusted for age, sex, ethnic, education, Townsend deprivation index quintile, body mass index, Charlson Comorbidity Index, lifestyle score. Two-sided statistical tests were conducted and bolded values are statistically significant (<0.05).

**Table S5** **Interactions between biological age (KDM-BA and PhenoAge)** **acceleration and sex in predicting incident CVD and** **premature mortality at follow-up**

**Ⅰ. Interaction test for incident CVD^a^**

| Sex | p-values of interaction |
| --- | --- |
| KDM-BA acceleration | <0.001 |
| PhenoAge acceleration | <0.001 |

Abbreviations: KDM-BA, Klemera-Doubal Method biological age; CVD, cardiovascular disease.

^a^Model was adjusted for age, sex, ethnic, education, Townsend deprivation index quintile, body mass index, Charlson Comorbidity Index, lifestyle score, and the interaction terms of biological age accelerations with sex. Two-sided statistical tests were conducted and bolded values are statistically significant (<0.05).

**Ⅱ. Interaction test for premature mortality^a^**

| Sex | p-values of interaction |
| --- | --- |
| KDM-BA acceleration | 0.596 |
| PhenoAge acceleration | <0.001 |

Abbreviations: KDM-BA, Klemera-Doubal Method biological age; CVD, cardiovascular disease.

^a^Model was adjusted for age, sex, ethnic, education, Townsend deprivation index quintile, body mass index, Charlson Comorbidity Index, lifestyle score, and the interaction terms of biological age accelerations with sex. Two-sided statistical tests were conducted and bolded values are statistically significant (<0.05).

**Table S6** **Associations of biological age (KDM-BA and PhenoAge)** **acceleration with incident CVD and premature mortality at follow-up among individuals with >2 years of follow-up (fully adjusted model)^a^**

|  | Incident CVD | | Premature mortality | |
| --- | --- | --- | --- | --- |
|  | Event/person-years | aHR (95% CI) | Event/person-years | aHR (95% CI) |
| KDM-BA acceleration (Continuous) | 22801/1040797 | **1.12 (1.1-1.13)** | 3179/1631518 | **1.01 (0.97-1.05)** |
| KDM-BA acceleration (Quartiles) |  |  |  |  |
| Q1 | 5242/290343 | Ref | 753/453861 | Ref |
| Q2 | 5331/274810 | **1.08 (1.04-1.12)** | 763/432981 | 1.00 (0.90-1.10) |
| Q3 | 5479/251296 | **1.14 (1.09-1.18)** | 754/393993 | 0.99 (0.89-1.10) |
| Q4 | 6749/224348 | **1.32 (1.27-1.37)** | 909/350683 | **1.06 (0.96-1.18)** |
| PhenoAge acceleration (Continuous) | 22801/1040797 | **1.1 (1.08-1.11)** | 3179/1631518 | **1.10 (1.06-1.14)** |
| PhenoAge acceleration (Quartiles) |  |  |  |  |
| Q1 | 4688/277778 | Ref | 671/437449 | Ref |
| Q2 | 5111/266601 | 1.00 (0.96-1.04) | 679/417442 | 0.97 (0.87-1.08) |
| Q3 | 5913/262907 | **1.06 (1.02-1.11)** | 778/412743 | 1.01 (0.91-1.12) |
| Q4 | 7089/233511 | **1.22 (1.17-1.27)** | 1051/363884 | **1.16 (1.04-1.28)** |

Abbreviations: KDM-BA, Klemera-Doubal Method biological age; aHR, adjusted hazard ratio; 95% CI, 95% confidence interval; CVD, cardiovascular disease; Q, quartile.

^a^Model was adjusted for age, sex, ethnic, education, Townsend deprivation index quintile, body mass index, Charlson Comorbidity Index, lifestyle score. Two-sided statistical tests were conducted and bolded values are statistically significant (<0.05).

**Table S7** **Associations of biological age (KDM-BA and PhenoAge)** **acceleration with incident CVD and premature mortality at follow-up by additionally controlling for whether individuals diagnosed with cancer during follow-up (fully adjusted model)^a^**

|  | Incident CVD | | Premature mortality | |
| --- | --- | --- | --- | --- |
|  | Event/person-years | aHR (95% CI) | Event/person-years | aHR (95% CI) |
| KDM-BA acceleration (Continuous) | 24281/1057040 | **1.09 (1.07-1.10)** | 3614/1780840 | **1.02 (0.99-1.06)** |
| KDM-BA acceleration (Quartiles) |  |  |  |  |
| Q1 | 5587/294957 | Ref | 852/496929 | Ref |
| Q2 | 5667/279005 | **1.06 (1.02-1.10)** | 863/471982 | 1.00 (0.91-1.10) |
| Q3 | 5851/255330 | **1.10 (1.06-1.14)** | 855/431041 | 1.01 (0.91-1.11) |
| Q4 | 7176/227748 | **1.24 (1.19-1.29)** | 1044/380888 | 1.09 (0.99-1.20) |
| PhenoAge acceleration (Continuous) | 24281/1057040 | **1.08 (1.07-1.10)** | 3614/1780840 | **1.13 (1.09-1.16)** |
| PhenoAge acceleration (Quartiles) |  |  |  |  |
| Q1 | 4975/294957 | Ref | 746/476703 | Ref |
| Q2 | 5399/279005 | 0.99 (0.95-1.03) | 757/454832 | 0.98 (0.88-1.08) |
| Q3 | 6301/255330 | **1.05 (1.01-1.09)** | 894/450099 | 1.04 (0.94-1.14) |
| Q4 | 7606/227748 | **1.18 (1.14-1.22)** | 1217/399206 | **1.20 (1.09-1.32)** |

Abbreviations: KDM-BA, Klemera-Doubal Method biological age; aHR, adjusted hazard ratio; 95% CI, 95% confidence interval; CVD, cardiovascular disease; Q, quartile.

^a^Model was adjusted for age, sex, ethnic, education, Townsend deprivation index quintile, body mass index, Charlson Comorbidity Index, lifestyle score. Two-sided statistical tests were conducted and bolded values are statistically significant (<0.05).

**Table S8** **Associations of biological age (KDM-BA and PhenoAge) acceleration with incident CVD and premature mortality at follow-up by using multiple imputation (fully adjusted model)^a^**

|  | Incident CVD | | Premature mortality | |
| --- | --- | --- | --- | --- |
|  | Event/person-years | aHR (95% CI) | Event/person-years | aHR (95% CI) |
| KDM-BA acceleration (Continuous) | 33774/2346657 | **1.11 (1.11-1.12)** | 4884/2346657 | **1.04 (1.03-1.06)** |
| KDM-BA acceleration (Quartiles) |  |  |  |  |
| Q1 | 7663/659826 | Ref | 1158/659826 | Ref |
| Q2 | 7847/620134 | **1.08 (1.07-1.10)** | 1153/620134 | 1.00 (0.96-1.04) |
| Q3 | 8303/569315 | **1.15 (1.13-1.17)** | 1167/569315 | 1.00 (0.97-1.04) |
| Q4 | 9961/497382 | **1.32 (1.30-1.34)** | 1406/497382 | **1.10 (1.06-1.15)** |
| PhenoAge acceleration (Continuous) | 33774/2346657 | **1.10 (1.10-1.11)** | 4884/2346657 | **1.16 (1.15-1.18)** |
| PhenoAge acceleration (Quartiles) |  |  |  |  |
| Q1 | 6915/631908 | Ref | 950/631908 | Ref |
| Q2 | 7553/600310 | 1.01 (0.99-1.02) | 1015/600310 | 1.03 (0.99-1.07) |
| Q3 | 8757/592712 | **1.07 (1.05-1.08)** | 1174/592712 | **1.06 (1.02-1.11)** |
| Q4 | 10549/521727 | **1.23 (1.22-1.25)** | 1745/521727 | **1.35 (1.30-1.40)** |

Abbreviations: KDM-BA, Klemera-Doubal Method biological age; aHR, adjusted hazard ratio; 95% CI, 95% confidence interval; CVD, cardiovascular disease; Q, quartile.

^a^Model was adjusted for age, sex, ethnic, education, Townsend deprivation index quintile, body mass index, Charlson Comorbidity Index, lifestyle score. Two-sided statistical tests were conducted and bolded values are statistically significant (<0.05).

**
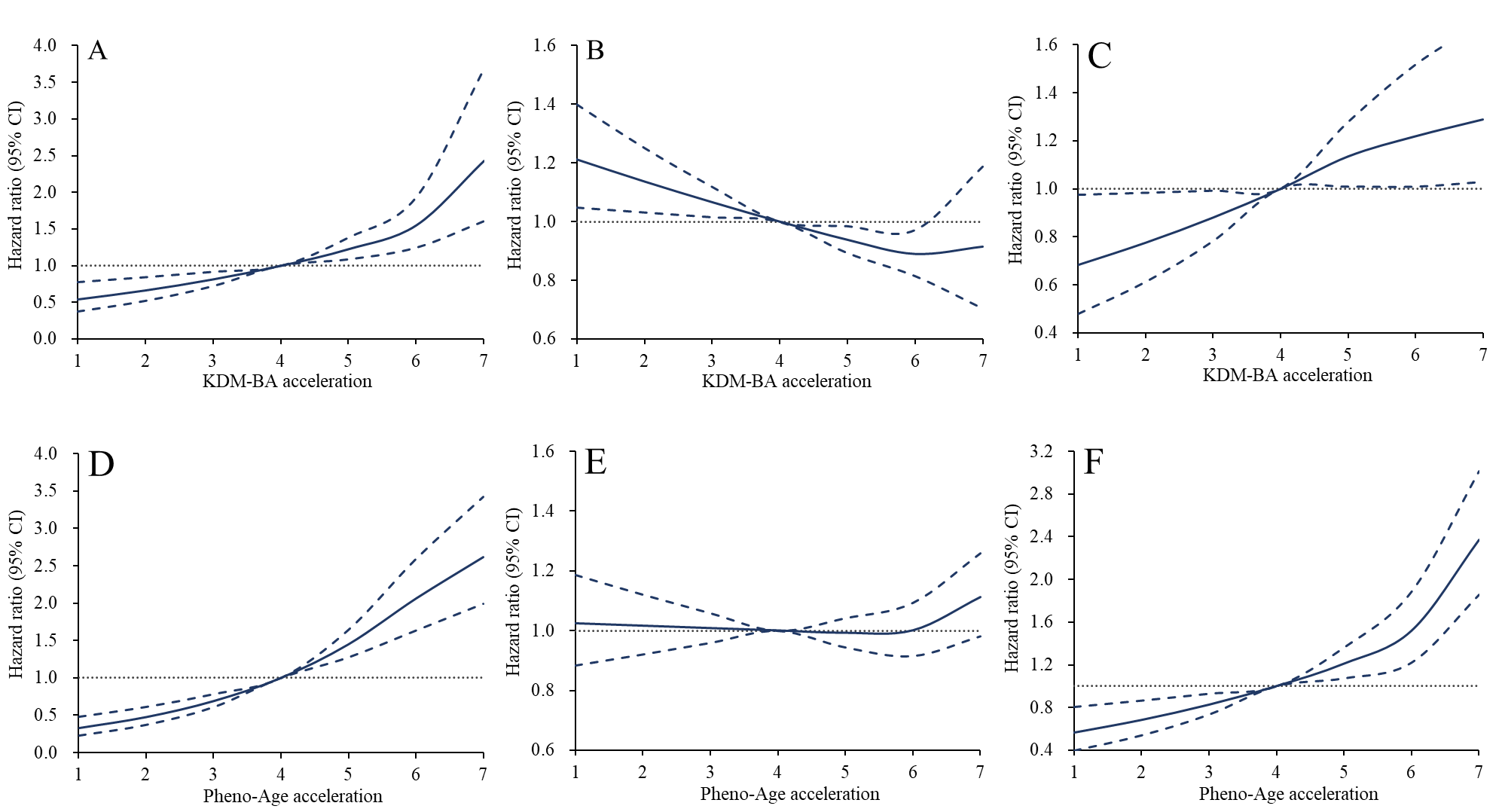
**

**Figure S1 Relationships of KDM-BA acceleration and PhenoAge acceleration with cause-specific premature mortality at follow-up. (A) KDMBA acceleration with premature death from respiratory disease, (B) KDMBA acceleration with premature death from cancer, (C) KDMBA acceleration with premature death from other causes, and (D) PhenoAge acceleration with premature death from respiratory disease, (E) PhenoAge acceleration with premature death from cancer, (F) PhenoAge acceleration with premature death from other causes.** Solid line: Point estimation; Dash line: Confidence limits. Restricted cubic spline regression model adjusted for age, sex, ethnicity, education, Townsend deprivation index quintile, body mass index, Charlson Comorbidity Index, and lifestyle score.
